# Last but not least: BFL-1 as an emerging target for anti-cancer therapies

**DOI:** 10.1042/BST20220153

**Published:** 2022-07-28

**Authors:** Gaoyuan Wang, Sarah T. Diepstraten, Marco J. Herold

**Affiliations:** 1 The Walter and Eliza Hall Institute of Medical Research, Melbourne, VIC, Australia; 2 Department of Medical Biology, University of Melbourne, Melbourne, VIC, Australia

**Keywords:** BCL-2 proteins, cancer, cell death, chemotherapy

## Abstract

BFL-1 is an understudied pro-survival BCL-2 protein. The expression of BFL-1 is reported in many cancers, but it is yet to be clarified whether high transcript expression also always correlates with a pro-survival function. However, recent applications of BH3-mimetics for the treatment of blood cancers identified BFL-1 as a potential resistance factor in this type of cancer. Hence, understanding the role of BFL-1 in human cancers and how its up-regulation leads to therapy resistance has become an area of great clinical relevance. In addition, deletion of the murine homologue of BFL-1, called A1, in mice showed only minimal impacts on the well-being of these animals, suggesting drugs targeting BFL-1 would exhibit limited on-target toxicities. BFL-1 therefore represents a good clinical cancer target. Currently, no effective BFL-1 inhibitors exist, which is likely due to the underappreciation of BFL-1 as a potential target in the clinic and lack of understanding of the BFL-1 protein. In this review, the roles of BFL-1 in the development of different types of cancers and drug resistant mechanisms are discussed and some recent advances in the generation of BFL-1 inhibitors highlighted.

## Introduction

**Figure 1.**
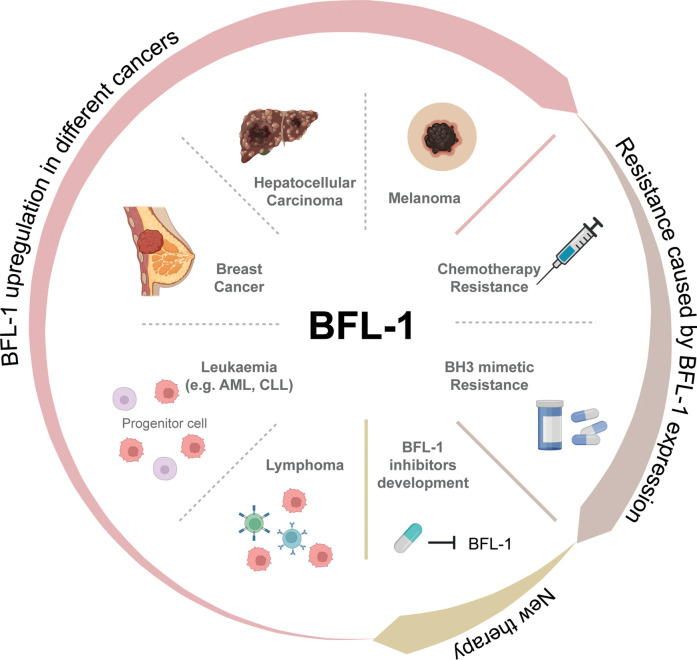
The role of BFL-1 in cancer.

The BCL-2 family proteins are key-regulators of the intrinsic apoptotic pathway. Members of this family contain BCL-2 homology (BH) regions and can be broadly separated into two groups: pro-survival proteins (BCL-2, MCL-1, BCL-XL, A1 (BFL-1 in humans) and BCL-W) and pro-apoptotic proteins. The pro-apoptotic proteins can be further divided into BH3-only proteins (BAD, BID, BIK, BIM, BMF, HRK, NOXA and PUMA) and BAX/BAK-like proteins [1]. The selective binding of pro-survival and pro-apoptotic proteins regulates the intrinsic apoptosis pathway and therefore controls cell survival. This pathway is activated in response to cellular stresses such as oncogene activation, DNA damage and cytokine/growth factor withdrawal [2]. In response to these stress signals, pro-apoptotic BH3-only proteins are induced, which in turn activate BAX and BAK, either through neutralisation of pro-survival proteins or direct activation. This leads to mitochondrial outer membrane permeabilization, the release of cytochrome c from the mitochondria into the cytosol, and ultimately the formation of the apoptosome. Altered expression of proteins in the apoptosis pathway, such as up-regulation of pro-survival proteins, can help cells evade cell death, thereby promoting tumorigenesis [3]. The pro-survival protein BFL-1 is a relatively understudied member of this family. However, in recent years, a role for BFL-1 in the survival and therapy resistance of diverse cancers has begun to be uncovered (Figure 1).

## BFL-1 and A1 discovery and function

In 1991, the murine *Bcl2a1* gene (which encodes the mouse homologue of human BFL-1, called A1) was first identified in a set of mRNAs which were up-regulated in bone marrow-derived macrophages stimulated with granulocyte-macrophage colony-stimulating factor [4]. Its relationship to the BCL-2 family of genes was not recognised until 1995, when mRNA of its human homologue was isolated from a fetal liver at 22 weeks of gestation and thus the gene was named ‘*Bcl-2 related gene expressed in fetal liver*’ (BFL-1) [5]. Due to its homology to other BCL-2 family genes, and its overexpression in clinical samples of stomach cancer, BFL-1 was hypothesised to promote cell survival during stomach tumorigenesis [5]. Following these discoveries, in 1996, it was found that BFL-1 can suppress p53-induced apoptosis like other Bcl-2 family members, clarifying its role as a pro-survival protein involved in apoptosis regulation [6]. Like overexpression of the other pro-survival proteins BCL-2, MCL-1, BCL-XL and BCL-W, overexpression of BFL-1 has been shown to accelerate MYC-driven myeloid leukemogenesis [7], as well as MYC-driven lymphomagenesis in the *Eµ-Myc* mouse model of B cell lymphoma [8].

In 1997, mouse *Bcl2a1* (encoding A1) was mapped to chromosome 9 and human *BCL2A1* (encoding BFL-1) was mapped to chromosome 15 [9,10]. Mouse *Bcl2a1* underwent three duplication events, resulting in three functional and identical paralogues (*Bcl2a1a*, *Bcl2a1b*, *Bcl2a1d*) and one pseudogene (*Bcl2a1c*) [11]. The functional A1 isoforms are differentially expressed in the haematopoietic compartment. High expression of A1-b was observed in resting T cells, B lymphocytes and thymocytes, while in CD8+ activated T cells, A1-a and A1-d are preferentially expressed [12]. In contrast to mouse *Bcl2a1,* only one copy of human *BCL2A1* is present in the genome. However, a short splicing variant of BFL-1, BFL-1S, was identified through reverse-transcriptase polymerase chain reaction (RT-PCR) analysis. In normal tissues, BFL-1S was detected predominantly in lymph nodes. In cancers, its expression was detected mainly in B-lymphoid leukaemia cells [13].

**Figure 2.**
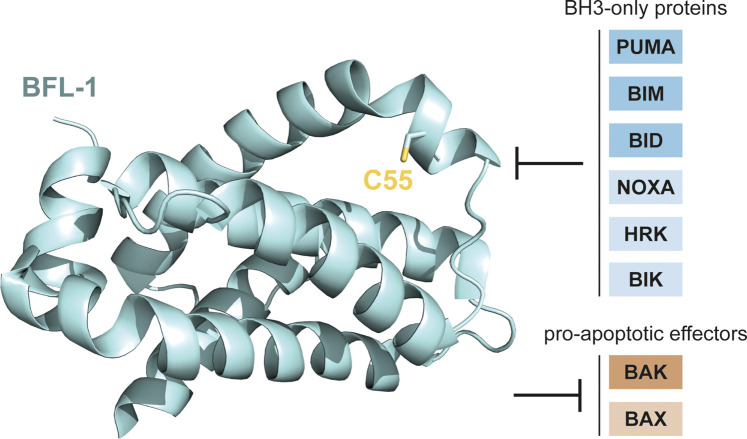
The crystal structure of BFL-1 showing the unique C55 residue in its BH3 binding groove (PDB ID: 5WHI) [21], and BFL-1's interacting protein partners. Structural image was generated using PyMOL (Version 2.5.0, Schrödinger, LLC).

Human BFL-1 and its mouse homologue A1 share all four BH-domains with BCL-2 and can form a groove which can be engaged by pro-apoptotic BH3-only proteins. *In vitro* binding studies and functional complementation experiments in cells have shown that BFL-1 can interact with the BH3-only proteins BIM, PUMA, NOXA, BIK, BID and HRK, as well as the pro-apoptotic effector proteins BAK and, to a lesser extent, BAX (Figure 2) [14–16]. Different from most BCL-2 family proteins, whose C-terminal portion localises them to intracellular membranes, the C-terminus of A1 does not function as a membrane anchor. Instead, it mediates the ubiquitination and degradation of A1 via the proteosome [17]. Similar to what has been found for mouse A1, the C-terminus of human BFL-1 does not contain a well-defined transmembrane domain. It is instead responsible for the regulation of BFL-1's anti-apoptotic function by enabling its rapid turnover under steady-state conditions (BFL-1 has a short half-life of less than an hour compared to other stable BCL-2 family proteins such as BCL-2 with a half-life of about 20 hours) [17–20]. The crystal structure of BFL-1 was first reported in complex with BIM in 2008 [14]. In recent years, the structure of unliganded BFL-1 was solved, allowing for a better understanding of its BH3 binding sites. A unique surface-exposed cysteine C55 was found to play an important role in BH3 binding [21] (Figure 2). Together, these studies and many others helped to characterise the structure and function of this important pro-survival protein BFL-1.

### The role of A1 in haematopoietic development

A role for A1 in haematopoietic development was hypothesised as increased expression was found in various blood cells. For example, while *Bcl-2*, *Mcl-1* and *Bcl-x* are not induced in early T cells, *Bcl2a1* expression is enhanced because of pre-T cell receptor (TCR) signalling [22]. When immature B cells transition from transitional type 2 to follicular B cells, *Bcl2a1* was up-regulated [23]. Similar expression changes were observed in myeloid development [24].

Deletion of all functional A1 isoforms was initially considered impossible due to their proximity and other gene regions between the isoforms. Therefore, initial studies of A1 *in vivo* were performed using shRNA tools [25]. The knockdown approach demonstrated a minor role for A1 during haematopoietic development, but this was not seen in the knockout of all functional A1 isoforms; neither at steady state nor upon immune challenge [26–28]. The discrepancies observed between the total knockout versus knockdown models are probably due to the stress induced by the expression of shRNAs in mice. Importantly, complete deletion of A1 in mice had no impact on embryonic development or the general wellbeing of the animals. These results therefore suggest that the side effects on normal cells of targeting the pro-survival BFL-1 protein are likely to be minimal.

## BFL-1 in solid tumours

### Melanoma

BFL-1 has been suggested to play a role in the survival of the aggressive skin cancer, melanoma. Gene expression profiling of 40 metastatic and 42 primary melanoma patient samples found that *BCL2A1* was overexpressed in metastatic samples [29]. Similarly, a study of spontaneous melanoma central nervous system metastasis in preclinical models showed that *BCL2A1* was significantly up-regulated in melanoma cell lines, and the overexpression of *BCL2A1* facilitated intracranial tumour growth [30]. Importantly, functional studies showed that knockdown of BFL-1 alongside MCL-1 caused cell death in melanoma cell lines, indicating that BFL-1 may not only be up-regulated in some melanomas, but play a role in their survival [31].

### Breast cancer

BFL-1 has also been implicated in promoting the survival of some breast cancers. RT-PCR analysis of 30 breast cancer samples revealed that the expression of *BCL2A1* was increased in advanced breast cancer compared to early cancers, suggesting that BFL-1 may serve as a contributory factor in cancer progression [32]. More recently, it was found that *BCL2A1* can be activated by the oncoprotein MUC1-C through an NF-κB/p65-mediated mechanism [33]. This pathway plays an important role in the induction of epithelial–mesenchymal transition in triple-negative breast cancer. Therefore, it is possible that BFL-1 is providing essential pro-survival activity in this process.

### Hepatocellular carcinoma

The role of BFL-1 in hepatocellular carcinoma (HCC), the most common form of liver cancer, has also been explored. High-throughput genome-wide microarrays of samples from 32 HCC patients revealed that up-regulation of *BCL2A1* was correlated with metastasis of HCC into lymph nodes [34]. Recently, a novel lncRNA PANTR1/miR-587/*BCL2A1* pathway which promotes HCC progression has also been identified [35]. Finally, functional assays suggest that BFL-1 plays a role in the resistance of hepatoma cells to apoptosis induced by apolipoprotein M, a liver lipoprotein which can supress migration, invasion, and proliferation of hepatoma cells [36].

## BFL-1 in blood cancers

### Chronic lymphocytic leukaemia

In B-cell chronic lymphocytic leukaemia (CLL), the overexpression of BFL-1 confers an apoptosis-resistant phenotype. In a study of 37 CLL patients, significantly higher BFL-1 levels were found in patients with no response to chemotherapy versus those who had a partial response [37]. BFL-1 up-regulation was also found in tumour-promoting cells residing in the CLL tumour microenvironment. The proportion of tumour associated neutrophils was higher in the peripheral blood of CLL patients compared to age-matched healthy donors. When neutrophils from healthy donors were cultured with CLL-conditioned media, they exhibited a longer lifespan which was correlated with a significant increase in BFL-1 levels [38].

### Acute myeloid leukaemia

BFL-1 has been proposed to play an anti-apoptotic role in acute myeloid leukaemia (AML). Wilms Tumour protein (WT1) is a transcription factor regulating the development of the myeloid lineage. The overexpression of WT1 in AML is correlated with poor survival, and co-expression of WT1 and BFL-1 was found in 12 of 15 primary poor-prognosis AML samples [39]. Promoter-reporter assays revealed that *BCL2A1* was a direct target gene of WT1 [39]. It has also been reported that BFL-1 may contribute to the survival of acute promyelocytic leukaemia cells which have been treated with all-trans retinoic acid (ATRA) therapy. ATRA is used to promote differentiation of accumulated promyelocytes and causes induction of BFL-1 via activation of the transcription factor PU.1 [40].

### B cell lymphoma

BFL-1 overexpression has been reported in many types of B cell lymphoma. Transcriptional profiling of primary mediastinal large B cell lymphomas identified increased levels of *BCL2A1* activated by NF-κB as a gene signature for this disease [41]. High levels of BFL-1 expression were also identified in the ‘OxPhos’ subset of diffuse large B cell lymphoma (DLBCL) patient samples, in which genes related to mitochondrial function were significantly enriched [42]. In another study using DLBCL cell lines, downregulation of BFL-1 using shRNAs caused cells to become more sensitive to apoptosis induced by Rituximab and chemotherapies [43]. While little is so far known about how BFL-1 up-regulation can occur in cancer cells, Akasaka et al. [44] identified a novel double hit lymphoma (DHL) in an 82-year-old woman with MYC and *BCL2A1* rearrangement. In this case study, the lymphoma cells exhibited high levels of *BCL2A1* expression and it was suggested that BFL-1 could play a role in the survival of these cancer cells similar to the role played by BCL-2 in the more common MYC/BCL-2 double-hit lymphomas [44]. Together, these results suggest that BFL-1 may play a particularly important role in DLBCL. *BCL2A1* overexpression was also found in many other non-Hodgkin's lymphomas such as mantle cell lymphoma and anaplastic large cell lymphoma (ALCL) [45]. In anaplastic lymphoma kinase positive ALCLs, BFL-1 was necessary for sustained growth and survival, as identified by gene expression profiling followed by functional RNAi screening [46].

### T cell leukaemia

Interference in the pre-TCR checkpoint can lead to the onset of immature leukaemias by allowing abnormal T cells to escape regulation [47]. The initial steps of the leukaemogenic process induced by human T cell leukaemia virus type 1 (HTLV-1) was explored in mice with humanised immune systems. It was found that HTLV-1 can express a transactivator protein, TAX, which can preclude the assembly of the pre-TCR and override the pre-TCR checkpoint. Enhanced transcription of NF-κB and *BCL2A1* was able to compensate for the absence of the pre-TCR [47]. It was proposed that the proliferation and survival advantages of these dysregulated T cells may result in the emergence of a malignant clone.

## BFL-1 in drug resistance

### CLL

CLL cells in lymph nodes are more resistant to apoptosis than those in the peripheral blood due to the survival-promoting signals they receive from the microenvironment. The up-regulation of *BCL2A1* was identified as one of the key mechanisms of BH3-mimetic drug resistance in CLL cells receiving these signals from fibroblast feeder layers and cytokines *in vitro* [48]. In this study, the inhibition of BFL-1 protein expression using siRNA resulted in significant resensitisation of CLL cells to BH3-mimetic drugs. Other studies showed similar results using CD40 crosslinking antibodies to mimic microenviromental signals. In the latter study, induction of BFL-1 together with BCL-XL and MCL-1 in the lymph node microenvironment contributed to apoptosis blockade, which resulted in resistance of CLL cells to various drugs, including the BH3-mimetic drug venetoclax [49]. A recent study demonstrated that combining BH3-mimetic drugs targeting BCL-2, BCL-XL, and MCL-1 still cannot reverse the resistance caused by CD40 stimulation in CLL cells [50]. Hence, it was speculated that BFL-1 could be acting as a resistance factor in these cells. In another study comparing expression of apoptosis-regulating genes between fludarabine-sensitive CLL cells and those which were resistant, *BCL2A1* was the most significantly enriched gene in the fludarabine-resistant cells [51].

### AML

In a comparative transcriptome analysis of 206 venetoclax-sensitive and -resistant AML specimens, *BCL2A1* was identified as the most differentially expressed gene which was enriched in the resistant group [52]. Similarly, an integrated analysis incorporating clinical characteristics, genetic profiling and venetoclax response data from primary AML patient samples determined that high *BCL2A1* expression was one of the most significant factors which predicted resistance to venetoclax [53]. To explore the factors influencing resistance of primary AML cells with D835 mutation in the tyrosine kinase domain (*FLT3*-ITD/D835) to tyrosine kinase inhibitors (TKIs), cap analysis gene expression technology was used to compare these cells to unmutated *FLT3*-ITD cells [54]. The results suggested that overexpression of *BCL2A1* attenuated the sensitivity of *FLT3*-ITD mutated AML to TKIs and venetoclax treatment. Functional studies confirmed that inhibiting *BCL2A1* through STAT5 inactivation using a type I TKI gilteritinib or a BET inhibitor blocking the BRD4 binding site at the *BCL2A1* promoter can alleviate TKIs and venetoclax resistance in FLT3-ITD/D835 mutated AMLs [54].

### Lymphoma

BFL-1 was shown to be up-regulated in MYC/BCL-2 DHL cell lines treated with venetoclax *in vivo* [55]. The use of the BET bromodomain inhibitor CPI203 to indirectly down-regulate BFL-1 expression synergised with venetoclax to kill DHL cell lines and primary patient samples more effectively. Similarly, examination of a panel of lymphoma cell lines found that BFL-1^+^ lymphomas were less sensitive to BH3-mimetics targeting MCL-1 and BCL-2 [56]. When cyclin-dependent kinase 9 (CDK9) inhibitors were applied to down-regulate both BFL-1 and MCL-1, an induction of apoptosis in BH3-mimetic–resistant lymphoma cells was observed, and regression of BFL-1^+^ DLBCL was achieved in patient-derived xenograft (PDX) models [56]. Recently, unbiased genome wide CRISPR activation screens revealed a dominant role for A1 in venetoclax resistance in a novel model of aggressive lymphoma [57]. Furthermore, high BFL-1 levels were reported in ALCL cell lines which are resistant to MCL-1 or BCL-XL inhibitors, and siRNA knockdown of *BCL2A1* induced apoptosis in drug resistant cells [58]. Finally, a study of 27 T cell lymphoma patients showed that previously chemotherapy-treated patients exhibited significantly higher levels of *BCL2A1* expression compared to untreated patients [59]. Together, these studies indicate that BFL-1 can guard the survival of therapy-resistant cells.

### Multiple myeloma

From a gene transcript expression analysis of eight primary multiple myeloma patient samples that relapsed after chemotherapy, *BCL2A1* was identified as the most frequently increased factor, suggesting its therapy resistant role in multiple myeloma [60].

### Melanoma

BFL-1 has also been found to be relevant to treatment resistance of solid cancers such as melanoma. The correlation between *BCL2A1* amplification, directly regulated via the oncogene MITF, and poor BRAF inhibitor sensitivity was found both in melanoma cell lines and patient samples [61]. Recently, BFL-1 was shown to block the killing of some melanoma cells treated with a combination of MCL-1 and BCL-XL inhibitors [62]. The results suggested that in certain melanoma cells, the pro-survival proteins BFL-1, MCL-1 and BCL-XL are all responsible for cell survival.

## BFL-1 inhibitors

### Indirect inhibitors

Considering the critical emerging roles for BFL-1 in the survival and therapy resistance of diverse cancer cells, a reliable inhibitor of BFL-1 is needed for further functional studies and clinical application. Inhibition of BFL-1 has been indirectly achieved using a variety of compounds which regulate BFL-1 expression at the transcriptional or post-translational levels. A pan-histone deacetylase inhibitor was shown to inhibit BFL-1 expression in DLBCL via decreasing expression of the transcription factor WT1 and increasing expression of NOXA, which can bind and inactivate BFL-1 [63]. The CDK9 inhibitor AZD4573 was also found to indirectly down-regulate BFL-1 in conjunction with MCL-1 in BH3-mimetic–resistant lymphoma cell lines and lead to *in vivo* tumour regressions in BFL-1^+^ DLBCL PDX models [56]. However, as this compound has dual inhibitory effects targeting both MCL-1 and BFL-1, its clinical application may be limited due to on-target toxicities associated with targeting of MCL-1 [3].

### Direct inhibitors

Due to the differences in mechanisms which mediate BFL-1 expression in different conditions and cell types, an indirect inhibitor may only be useful in some scenarios. A direct inhibitor of BFL-1 is required for more widespread clinical use. To identify potential direct inhibitors of BFL-1, a screening campaign with a computational peptide library was performed to study the binding of BFL-1 to BH3-mimicking peptides [64]. Several high-affinity BFL-1 binders were identified and one of these peptides, FA1, was found to have a very slow dissociation rate from BFL-1 compared to other BCL-2 family proteins. Another approach to design peptides which bind to BFL-1 with high affinity was more recently demonstrated using recurring tertiary structural motifs [65].

Recently, the unique C55 residue in the BH3 binding groove of BFL-1 was identified and small molecule inhibitors were designed to target this residue [21]. Screening of a stapled peptide library generated a lead cysteine reactive NOXA construct [66]. Competitive fluorescence polarisation binding analyses showed that this construct can selectively target BFL-1. In a disulfide tethering screen, a small molecule called 4E14 was shown to effectively block the BFL-1 BH3 binding groove through covalent targeting of C55 [67]. Increased cytochrome c release was observed when mitochondria were treated with a BFL-1 expression construct together with 4E14, compared to the BFL-1 expression construct alone, indicating that 4E14 can overcome BFL-1 suppression of mitochondrial apoptosis. Another study screened an in-house compound library and identified ZH97 which can specifically bind to the C55 residue, block BFL-1/PUMA protein interactions, and induce apoptosis in BFL-1^+^ blood cancer cell lines [68].

## Conclusion

With each new vulnerability of cancer targeted and exploited, it seems that new complications emerge. In a not insignificant number of patients, we believe the increased levels of BFL-1 that have been detected in various types of tumours indicate the strong potential of targeting BFL-1 for the treatment of these pathologies. However, a common theme emerging from studies of normal development in the presence and absence of A1 is that mRNA levels of *Bcl2a1* do not necessarily predict the requirement of A1 for cell survival. Therefore, in preparation for employing specific and effective BFL-1 inhibitors in the clinic, functional studies to confirm the sensitivity of particular cancer types to loss of BFL-1 are increasingly important. The potential of this area of research is made even more exciting due to the well-established apparent lack of a requirement for A1 in the healthy, normal tissues of the mouse. The ability to safely inhibit BFL-1 in patients without side-effects could be a game changer for the treatment of certain types of cancer. BFL-1 might be the last of the BCL-2 family to be well characterised, but we are confident it will have been worth the wait.

## Perspectives

- BFL-1 plays no role in healthy cells, but high expression might contribute to tumour survival. In addition, BFL-1 was identified as the dominant resistance factor for several anti-cancer therapies.
- A1 knockout mouse models showed a minimal impact on the growth or survival of the animals, which suggests that targeting BFL-1 in humans will lead to very few on-target side effects.
- A better understanding of the mechanisms governing BFL-1 up-regulation in therapy resistance will benefit the development of direct and indirect BFL-1 inhibitors.

## Abbreviations

ALCL: anaplastic large cell lymphoma
AML: acute myeloid leukaemia
ATRA: all-trans retinoic acid
BFL-1: Bcl-2 related gene expressed in fetal liver
BH: BCL-2 homology
CDK9: cyclin-dependent kinase 9
CLL: chronic lymphocytic leukaemia
DHL: double hit lymphoma
DLBCL: diffuse large B cell lymphoma
HCC: hepatocellular carcinoma
HTLV-1: human T cell leukaemia virus type 1
PDX: patient-derived xenograft
RT-PCR: reverse-transcriptase polymerase chain reaction
TCR: T cell receptor
TKIs: tyrosine kinase inhibitors
WT1: Wilms Tumour protein
